# Editorial: Insights in extreme microbiology: 2022

**DOI:** 10.3389/fmicb.2023.1324080

**Published:** 2023-10-31

**Authors:** Andreas P. Teske, Virginia P. Edgcomb

**Affiliations:** ^1^Department of Earth, Marine and Environmental Sciences, University of North Carolina at Chapel Hill, Chapel Hill, NC, United States; ^2^Department of Geology and Geophysics, Woods Hole Oceanographic Institution, Woods Hole, MA, United States

**Keywords:** microbiology, insights, Research Topic, microbial community, 2022

Taking stock of the contributions to the “*Insights in extreme microbiology: 2022*” Research Topic again reveals the extraordinary breadth that exists within the field of Extreme Microbiology.

The strong showing of hydrothermal microbiology in the 2021 Research Topic continues this year. Two papers (Truong et al.; Adam-Beyer et al.), address hydrothermal vent microbiology from distinct biogeochemical/mineralogical and ecosystem angles. Truong et al. examine the formation and composition of pyrite spherules as a potential biomarker for the activity of omnipresent sulfur-reducing Thermococcales spp. at vent sites; and Adam-Beyer et al. examine linkages between microbial communities and particular niches on understudied Indian Ocean mid-ocean ridges, and they demonstrate the key role of hydrogen-oxidizing Epsilonproteobacteria.

Applied environmental research is represented by two papers, a genomic analysis of groundwater microbial communities in a continuous flow-through reactor that respond to phenol pollution (Yavari-Bafghi et al.), and bacterial community changes during artisanal indigo reduction under different (including highly alkaline) environmental conditions (Farjana et al.). These studies serve as helpful reminders that extreme microorganisms and conditions can be found in applied contexts and in traditional practices/procedures, far from well-known and charismatic field sites. First and foremost, microbes “see” and react to the chemistry of their immediate surroundings.

A study of spatially compressed microbial stratification within a cyanobacterial mat (Bourhane et al.), contrasts with an investigation of microbial methane cycling in extensive deep marine subsurface sediments in the Peru Trench (Lever et al.), and shows how biogeochemical gradients—from the micrometer scale in the hypersaline mat to the 100-meter scale in deep-sea sediments—shape the habitat, diversity and activity of microorganisms. Ecosystems with chemical gradients that microorganisms can exploit are particularly instructive, as they demonstrate the feedbacks that exist between changing geochemistry and microbial community structure and activity.

A particular theme from the 2021 Research Topic is continued here, in 2022, which is the pure culture-based physiological study on cesium ion resistance in *Microbacterium* sp. (Ishida et al.) which sheds light on microbial ecophysiology of heavy metal resistance, and its specific mechanisms at the single-cell level.

Finally, we are very happy to announce three creative and timely reviews in the 2022 topic. The first is on experimental simulations of early Earth's microbial activity, and presents strategies to ground-truth inferences on how microbial life has shaped the geochemical evolution of early Earth (Zhao et al.). The second is an introduction to the aeromicrobiome, which is the Earth's outermost microbial biosphere; this survey of a newly emergent research area attracted almost 2,000 views at the time of writing this editorial (Amato et al.). Finally, our most frequently viewed paper in this Research Topic (almost 5,000 views at the time of writing this editorial), is a review on decoding microbial dark matter by new approaches in extremophile cultivation (Schultz et al.). It was a distinct editorial pleasure to guide the gradual development of this review, and to help sharpen its focus.

Readers may be interested to take a look at the geographic origin of the contributions. We have papers from French, Spanish, and German working groups, from a widely dispersed German-American collaboration, from a Chinese group with links to England, from two Japanese labs with highly specific expertise, from an Iranian collaboration that has entrained support from the Iranian diaspora in Sweden, and—in the case of our high-impact authors—from Spanish and Brazilian microbiologists working out of KAUST in Saudi Arabia. Clearly, the old notion that environmental and extreme microbiology thrives best in European countries with a damp, clammy climate, and in midwestern land-grant universities where ruminants and C4-plants have center stage, no longer holds; we see instead emerging global patterns of expertise and authorship. That said, we are looking forward to more representation from Africa and Latin America, which do not suffer from a shortage of extreme microbial ecosystems.

Without further ado, here is the harvest of Extreme Insights for 2022.

## Author contributions

AT: Writing—original draft. VE: Writing—review & editing.

